# Never-smokers and the fraction of breast cancer attributable to second-hand smoke from parents during childhood: the Norwegian Women and Cancer Study 1991–2018

**DOI:** 10.1093/ije/dyab153

**Published:** 2021-08-01

**Authors:** Inger T Gram, Arne Bastian Wiik, Eiliv Lund, Idlir Licaj, Tonje Braaten

**Affiliations:** Department of Community Medicine, Faculty of Health Sciences, University of Tromsø (UiT) The Arctic University of Norway, Tromsø, Norway

**Keywords:** Avoidable breast cancer, breast-cancer prevention, breast-cancer risk, childhood exposure, cohort studies, environmental tobacco smoke, Norwegian Women and Cancer Study, passive smoking, population attributable fraction, second-hand smoke

## Abstract

**Background:**

Second-hand smoke (SHS) is not an established risk factor for breast cancer. We examined exposure to SHS from parents during childhood and breast-cancer risk overall and by oestrogen- and progesterone-receptor status in the Norwegian Women and Cancer Study. Furthermore, we utilized our nationally representative prospective cohort study to estimate the fraction of breast cancer attributable to parental SHS during childhood.

**Methods:**

We followed 45 923 never-smoking women, aged 34–70 years, who completed a baseline questionnaire between 1991 and 2007 through linkages to national registries through December 2018. We used Cox proportional-hazards models to estimate age-adjusted hazard ratios (HRs) and 95% confidence intervals (CIs). We estimated the attributable and the population attributable fraction of breast cancer with 95% CIs.

**Results:**

During a mean follow-up of 19.8 (6.8) years, 2185 women developed invasive breast cancer, confirmed by histology. Women exposed to SHS from parents during childhood had an 11% higher (95% CI: 1.02–1.22) risk of breast cancer compared with those who were not. No difference was found for oestrogen (*P*_heterogeneity_ = 0.31) and progesterone (*P*_heterogeneity_ = 0.95) receptor status. For women exposed, the attributable fraction was 10.3% (95% CI: 1.8–18.0), whereas the population attributable fraction of breast cancer was 7.0% (95% CI: 1.0–13.0).

**Conclusions:**

Our results suggest that 1 in 14 breast-cancer cases could have been avoided in the absence of SHS exposure from parents during childhood in a population of never-smoking women. The cancer burden attributable to SHS may be underestimated.

Key Messages

- Worldwide, <10% of women are daily smokers, whereas a high proportion are exposed to second-hand smoke (SHS) (i.e. passive or environmental tobacco smoke) during childhood.
- Our main finding suggests that 1 in 14 breast-cancer cases could have been avoided in the absence of SHS exposure from parents during childhood in a population of never-smoking women.
- The burden of breast cancer attributable to SHS exposure from parents estimated in the present study supports the notion that breast-cancer research and prevention efforts should have a lifetime approach.
- Public health agencies that have not recognized SHS as an established risk factor for breast cancer should reconsider the available evidence and update their conclusions.
- The World Health Organization Framework Convention on Tobacco Control, Article 8, protection from exposure to tobacco smoke, should be fully implemented and enforced globally.

## Introduction

Globally, female breast cancer is now the most commonly diagnosed cancer with an estimated 2.3 million new cases (11.7%) in 2020.^1^ In Norway, the breast-cancer incidence rate has doubled from 1959 to 2018.^2^ During the last century, the prevalence of daily smoking peaked for Norwegian men during the late 1950s. Among women, the peak was lower and occurred 20 years later.^3^ In 1973, 1990 and 2018, the proportion of daily smokers were for men 52%, 37% and 12%, respectively. For women, the corresponding figures were 32%, 33% and 11%.^4^

Worldwide, ∼25% of men and 5% of women were daily smokers in 2015.^5^ In most countries, an estimated 15–50% of the population is exposed to second-hand smoke (SHS) (i.e. passive or environmental tobacco smoke), whereas in some countries, SHS exposure affects as much as 70% of the population.^6^

Whereas active smoking is an emerging risk factor for breast cancer, the evidence is more inconsistent for exposure to SHS and risk of breast cancer.^7–11^ Two reviews^12^^,^^13^ and two meta-analyses,^14^^,^^15^ published subsequently to the four expert reports,^7–10^ note that the evidence has increased for a moderately higher breast-cancer risk caused by exposure to SHS.

We have previously reported that women who were ever-smokers or never-smokers exposed to SHS both had a higher risk of breast cancer compared with those not exposed.^16^

As pointed out by Jha,^17^ the full effects of smoking can take ≤50 years to measure in individuals and ≤100 years to measure in a population. We wanted to examine SHS exposure from parents during childhood in a population of never-smokers and the risk of breast cancer overall, and by oestrogen- and progesterone-receptor statuses. We also wanted to estimate the fraction of breast cancer attributable to this exposure among those exposed and in the population of never-smoking women.

## Methods

### Study design and participants

The Norwegian Women and Cancer Study cohort profile has been previously described in detail.^18^^,^^19^ Briefly, the Central Population Register selected a random sample of women according to year of birth. Subsequently, an invitation to participate in the study, with a baseline questionnaire and a pre-stamped return envelope enclosed, was mailed to each woman. The National Data Inspectorate and the Regional Committee for Medical and Health Research Ethics approved the study. All women gave informed consent.

Women who completed a questionnaire during three waves of data collection, namely 1991–1992, 1996–1997 and 2003–2007 (*n *=* *172 478), were the baseline population. The overall response rate was 52.7%. We excluded women with prevalent cancer (*n *=* *6666), who had emigrated (8), who died before start of follow-up (11), who were born after 1957 (*n *=* *3169), who had missing information or were ever-smokers (*n *=* *107 083) or had missing information on exposure to SHS from parents during childhood (*n *=* *9602). Altogether, 8542 women with breast cancer were excluded in this process. The analytical cohort comprised the 45 923 remaining women.

### Data collection

The baseline questionnaires elicited information about years of education, current height and weight for the calculation of body mass index (BMI, kg/m^2^), reproductive and lifestyle factors. Most questionnaires asked about exposure to SHS during childhood, although in two slightly different versions: ‘Did any of the adults smoke at home when you were a child?’ (yes, no). If, yes, ‘Mother, father, both, others’; or ‘Did any of your parents smoke at home when you were a child?’ (yes, no). If yes, ‘How many cigarettes did they smoke per day in total?’ Questions about the intensity of SHS exposure (number of cigarettes smoked by the parents) were only asked in a few questionnaires and not considered in this analysis. Almost half of the women were asked at enrolment about current SHS exposure at home from a partner. We dichotomized the women as exposed to SHS by parents during childhood or not. The latter group is the reference group throughout the paper.

We followed the women through linkages to the Cancer Registry of Norway and the Norwegian Central Population Register to identify all cancer cases, emigrations and deaths, respectively, using the unique national 11-digit personal identification number. We classified breast-cancer cases according to the organ site code (C50) in the International Classification of Diseases, Tenth Revision, and according to oestrogen and progesterone tumour-receptor-status categories [ER-positive (ER+), ER-negative (ER–), PR+, PR–] based on information from the registry. We calculated person-years from the start of follow-up to the date of any incident cancer diagnosis (except basal cell carcinoma), emigration, death or the end of follow-up (31 December 2018), whichever came first.

### Statistical analysis

We calculated percentages (%) or means with standard deviation (± SD) for the distribution of selected characteristics of the study population. We estimated crude breast-cancer incidence rates overall and according to the SHS-exposure group, by dividing the number of cases by the total number of person-years. We then age-adjusted the rates to the world standard population (http://seer.cancer.gov/stdpopulations/).

We used the Cox proportional-hazards regression model with age as the underlying timescale to estimate age-adjusted hazard ratios (HRs) and 95% confidence intervals (CIs) for the association between exposure to SHS from parents during childhood and risk of breast cancer overall, and according to oestrogen and progesterone hormone-receptor statuses. Subsequently, we performed competing risk analysis using cause-specific hazard models for time to hormone-receptor-status breast-cancer outcomes, with censoring at diagnosis for any breast-cancer cases with a receptor status other than that being considered.^20–22^ The receptor-status outcomes considered were ER+ and ER–, PR+ and PR–, and the four combinations. Cases with missing information on ER status (*n *=* *374), PR status (*n *=* *433) or both (*n* = 10) were excluded from the corresponding analyses. Heterogeneity in risk by SHS between the tumour hormone-receptor-status categories was assessed using a Wald test.

We examined whether each of the following selected covariates of birth cohort, education level, age at menarche, a combination of age at first birth and parity, BMI, physical activity, alcohol consumption or menopausal status changed the HR estimate by >5%. None of them did.

We present age-adjusted HR estimates throughout the paper. Using the Wald test, we tested for heterogeneity in the SHS—breast cancer association by eight [birth cohort (<1950, ≥1950), education (<13, ≥13), age at menarche (<12, ≥12), parous (yes, no), BMI (<25 kg/m^2^, ≥25 kg/m^2^), physical-activity score (<4, ≥4), alcohol consumption (yes, no) all at enrolment, and menopausal status (pre, post), changing to postmenopausal status during follow-up with available information or at age 51 years, whichever came first] selected factors. The proportional-hazards assumption was tested using Schoenfeld residuals and was found to hold.^23^^,^^24^ We performed one sensitivity analysis restricted to never-smokers with information on both SHS exposures, and another that included the entire cohort of ever- and never-smokers. In both, the estimates for SHS exposure during childhood and risk of breast cancer stayed materially the same (data not shown).

We estimated the attributable fraction (AF) and the population attributable fraction (PAF) to indicate the proportion of the breast-cancer cases that could have been avoided in women exposed and in never-smokers in the absence of SHS exposure during childhood. We used the formula $\text{PAF}=\frac{Pe\times (\text{RRe}-1)}{Pe\times \text{RRe}+(1-\text{Pe})}$, where the notation *P*e is the proportion of persons in the population exposed to the risk factor and *RR*_e_ is the relative risk in the exposed compared with the unexposed group (World Health Organization, 2012). We calculated two-sided 95% CIs for the AFs and PAFs using the PUNAF Stata module.^25^ We performed the analyses using STATA version 16.0 (Stata Corp, College Statistics, TX, USA).

## Results

At enrolment, the mean age of the never-smokers was 49.8 years. Altogether, 66.0% (*n *=* *30 471) reported having been exposed to SHS from parents during childhood. During 911 085 person-years of observation (mean follow-up time was 19.0 years), we ascertained 2185 incident cases of primary invasive breast cancer, confirmed by histology. The age-standardized incidence rate for breast cancer was 234.5—overall, 243.2—for those exposed and 217.4 per 100 000 person-years for those not exposed.

Table 1 shows that women who reported exposure to SHS from parents during childhood were younger at enrolment, at breast-cancer diagnosis, at menarche, at menopause and at first childbirth; they were less educated; and more were consuming alcohol and had a higher average alcohol consumption compared with those not exposed (Table 1).

**Table 1.** Selected characteristics of never-smokers at enrolment, overall and by exposure to second-hand smoke by parents during childhood, the Norwegian Women and Cancer Study, 1991–2018 *(n *=* *45 923)

Characteristics	Never-smokers	Second-hand smoke exposure		*P*-value^a^
	Total	No	Yes	
	*n* = 45 923	*n* = 15 452	*n* = 30 471	
Age at enrolment (s.d.)	49.8 (8.4)	50.4 (8.7)	49.5 (8.3)	0.00
Age at diagnosis (s.d.)	61.6 (8.5)	62.2 (8.6)	61.3 (8.4)	0.03
Person-years of follow-up (s.d.)	19.8 (6.8)	19.9 (6.7)	19.8 (6.9)	0.03
Primary invasive breast cancers (*n*)	2185	692	1493	0.05
Family history of breast cancer (%)	5.6	1.8	3.7	0.81
Education ≥13 years (%)	49.7	51.7	48.7	0.00
Age at menarche (years) (s.d.)	13.3 (1.4)	13.3 (1.4)	13.3 (1.4)	0.00
Postmenopausal (%)	50.5	52.2	49.7	0.00
Age at menopause (s.d.)	49.2 (4.7)	49.4 (4.5)	49.1 (4.7)	0.00
Parous women (%)	90	89	90	0.00
Number of children (s.d.)	2.3 (1.3)	2.4 (1.4)	2.26 (1.2)	0.00
Age at first childbirth (s.d.)	24.7 (4.3)	25.1 (4.3|)	24.6 (4.3)	0.00
Body mass index (kg/m^2^) (s.d.)	24.4 (4.0)	24.2 (3.8)	24.4 (4.0)	0.00
Physical-activity score^b^ (s.d.)	5.8 (1.8)	5.8 (1.8)	5.7 (1.9)	0.01
Teetotallers (%)	18.2	26.0	14.2	0.00
Alcohol consumption^c^ (g/day) (s.d.)	2.5 (3.9)	2.0 (3.9)	2.8 (4.0)	0.00

a *T*-test or chi-square test for differences between SHS exposure from parents or not.

b Physical-activity score in 10 categories (recreational and work-related).

c Among drinkers.

s.d., standard deviation.

Women exposed to SHS from parents during childhood had an 11% higher (95% CI: 1.02–1.22) risk of breast cancer compared with those not exposed. Table 2 shows that the age-adjusted HRs for ER+ tumours was 17% higher (95% CI: 1.05–1.30), for PR+ tumours 10% higher (95% CI: 1.02–1.20), for ER– tumours 8% lower (95% CI: 0.70–1.20) and for PR– tumours 16% (95% CI: 0.96–1.40) higher compared with the corresponding reference group. No difference was found for oestrogen (*P*_heterogeneity_ = 0.31) and progesterone (*P*_heterogeneity_ = 0.95)-receptor status (Table 2).

**Table 2.** Age-adjusted hazard ratios (HRs) and 95% confidence intervals (CIs) for ER+, ER–, PR+, PR– breast-cancer cases (*n *=* *2175)^a^ according to second-hand smoke (SHS) exposure from parents during childhood, the Norwegian Women and Cancer Study, 1991–2018

SHS exposure	ER-positive		ER-negative		*P*_heterogeneity_ ^b^	PR-positive		PR-negative		*P*_heterogeneity_ ^b^
	(*n* = 1580)		(*n* = 231)			(*n* = 1232)		(*n* = 520)		
	Cases	HR	Cases	HR		Cases	HR	Cases	HR	
		(95% CI)		(95% CI)			(95% CI)		(95% CI)	
No	487	1.00 (ref)	84	1.00 (ref)		384	1.00 (ref)	161	1.00 (ref)	
Yes	1093	1.17	147	0.92	0.31	848	1.10	359	1.16	0.95
		(1.05–1.30)		(0.70–1.20)			(1.02–1.20)		(0.96–1.40)	

a After excluding 10 cases with missing data on both ER and PR status.

b *P* for heterogeneity between receptor status in a competing risk model.

Similar results were found for the combination of positive and negative hormone-receptor statuses [ER+/PR+ tumours 10% higher (95% CI: 1.01–1.20), ER+/PR– tumours 7% higher (95% CI: 0.98–1.18), ER–/PR+ tumours 11% higher (95% CI: 1.02–1.22) and ER–/PR– tumours 13% higher (95% CI: 1.03–1.24) compared with the corresponding reference group].

Table 3 shows that the age-adjusted HR estimates for breast cancer overall did not differ according to the eight selected variables (Table 3). The AF of breast cancer was 10.3% (95% CI: 1.8–18.0) in the women exposed to SHS from parents during childhood and the PAF of breast cancer was 7.0% (95% CI: 1.0–13.0) in the population of never-smokers.

**Table 3.** Age-adjusted hazard ratios (HRs) and 95% confidence intervals (CIs) by exposure to second-hand smoke (SHS) from parents during childhood according to selected characteristics, the Norwegian Women and Cancer Study, 1991–2018 (*n *=* *45 923)

Characteristics	SHS	Number	Age-adjusted	*P*_heterogeneity_ ^b^
	exposure	of cases	HR (95% CI)	
		(*n* = 2185)^a^		
Year of birth (*n* = 2185)				
<1950	No	439	1.00 (Ref.)	
	Yes	888	1.12 (0.99–1.25)	
≥1950	No	253	1.00 (Ref.)	
	Yes	605	1.11 (0.96–1.28)	0.94
Education(*n* = 2113)^a^				
<13 years	No	312	1.00 (Ref.)	
	Yes	735	1.18 (1.03–1.34)	
≥13 years	No	364	1.00 (Ref.)	
	Yes	702	1.06 (0.93–1.19)	0.25
Age at menarche (*n* = 2152)^a^				
<12 years	No	48	1.00 (Ref.)	
	Yes	130	1.18 (0.85–1.64)	
≥12 years	No	639	1.00 (Ref.)	
	Yes	1335	1.10 (1.00–1.21)	0.70
Parity (*n* = 2185)				
Nulliparous	No	106	1.00 (Ref.)	
	Yes	182	1.04 (0.82–1.32)	
Parous	No	586	1.00 (Ref.)	
	Yes	1311	1.13 (1.03–1.25)	0.49
Body mass index^b^^,^^d^ (*n* = 2147)				
<25 kg/m^2^	No	456	1.00 (Ref.)	
	Yes	964	1.12 (1.06–1.26)	
≥25 kg/m^2^	No	232	1.00 (Ref.)	
	Yes	495	1.10 (0.95–1.28)	0.83
Physical-activity score^a^^,^^c^(*n* = 2029)				
<4	No	60	1.00 (Ref.)	
	Yes	163	1.26 (0.94–1.69)	
≥4	No	580	1.00 (Ref.)	
	Yes	1226	1.10 (1.00–1.21)	0.39
Alcohol consumption^a^ (*n* = 2092)				
Non-drinkers	No	160	1.00 (Ref.)	
	Yes	203	1.17 (0.96–1.44)	
Drinkers	No	497	1.00 (Ref.)	
	Yes	1232	1.09 (0.98–1.21)	0.51
Menopausal status^a^^,^^d^ (*n* = 1939)				
Premenopausal	No	360	1.00 (Ref.)	
	Yes	763	1.04 (0.92–1.18)	
Postmenopausal	No	260	1.00 (Ref.)	
	Yes	556	1.22 (1.05–1.41)	0.12

a Some totals are <2185 due to missing numbers.

b Wald test for heterogeneity.

c Physical-activity score in 10 categories (recreational and work-related)—Low PA <4.

d Changing to postmenopausal status during follow-up with available information or at age 51 years, whichever came first.

## Discussion

To our knowledge, our study is the first to estimate the fraction of breast cancer in never-smokers attributable to exposure to SHS from parents during childhood. Compared with those not exposed, we observe a higher breast-cancer risk for women exposed. This higher risk seems to be of a similar magnitude for the different tumour hormone-receptor types. Also, the magnitude of the higher breast-cancer risk was consistent when we stratified by eight selected variables. Finally, we estimated that in women exposed, 1 in 10 and, in the population of never-smokers, 1 in 14 breast-cancer cases could have been avoided in the absence of SHS from parents during childhood. These numbers are of a magnitude that can explain some of the global increase in breast-cancer incidence.

In our 2016 report, the majority (65%) of women were ever-smokers. We estimated that the fraction of breast cancer in Norway attributable to ever-smoking was 12%, whereas it was 3% for any SHS exposure.^16^ In the present study, the majority (66%) of never-smokers were exposed to SHS during childhood. The present results show that the fraction of breast cancer attributable to SHS exposure from parents during childhood in the population of never-smoking women was of the same magnitude as ever-smoking was in the entire Norwegian female population.

Among the seven cohort studies,^26–32^ with >500 breast-cancer cases among never-smokers and information on parental SHS exposure during childhood, only one showed a higher risk of breast cancer. This was revealed for exposure *in utero* (16%) and during childhood/adolescence from birth to 18 years (17%). This report from the Sister cohort study conducted in the USA and Puerto Rico did not find an association with either active or adult SHS exposure and breast-cancer risk. All analyses concerning SHS exposures were restricted to never-smoking women.^32^ In the present study, we used a dichotomous response to the question of childhood SHS exposure without defining childhood. Nevertheless, our results are similar to those from the Sister study, which had detailed questions related to the timing and duration of SHS exposure. However, these responses were dichotomized into two (definitely/probably ‘yes’ and definitely/probably ‘no’) exposure categories.^32^

The reports within the UK Million Women Study^26^ and the US California Teachers study^27^ included only never-smokers. The UK report^26^ asked about exposure from parents at 0 and 10 years, whereas the US report asked about the duration and intensity of SHS exposure before age 20 years.^27^ The report from the Women’s Health Initiative Observational Study of postmenopausal women also asked about the duration of SHS exposure from parents during childhood (<18 years old).^29^

All but two^27^^,^^29^ of the cohort studies ended up with dichotomous categories for SHS exposure during childhood^26^^,^^28^^,^^32^ or for any SHS exposure^16^^,^^30^^,^^31^ in the main analyses.

For the cohorts that gave information on SHS exposure during childhood in never-smokers, compared with our study, the proportion was lower in two^28^^,^^29^ and higher in three.^26^^,^^27^^,^^32^

Results from 13 cohort studies^16^^,^^28^^,^^30^^,^^31^^,^^33–41^ suggest that women smoking actively before the breast tissue is fully matured may be especially susceptible for developing breast cancer. Nine of these studies^33–41^ did not have information on SHS. Most likely, the results are attenuated since women exposed to SHS are included in the reference groups. This is supported by four other cohort studies^16^^,^^30–32^ in which the association between active smoking and breast-cancer risk became stronger when women exposed to SHS were excluded from the respective reference groups.

In 2008, Johnson and Glantz noted that the evidence from epidemiological studies on SHS and risk of premenopausal breast cancer was in 2005 stronger than that for SHS and lung cancer in 1986.^42^ In their 2014 update, the two authors conclude that both active and passive smoking increase the risk of breast cancer.^12^ When Macacu *et al.*, in their meta-analyses, included only the 11 cohort studies, women exposed to SHS had a 7% higher risk of breast cancer overall compared with those who were not.^14^ In an update of a review on the association between breast cancer and the environment, the authors note that even though women exposed to SHS receive a much lower dose of carcinogens than active smokers do, both exposures seem to increase breast-cancer risk by about the same amount.^13^ Another meta-analyses found that SHS may increase the overall risk of cancer for never-smokers, and particularly the risk for breast cancer.^15^

One major strength of this study is that we have a high number of incident breast-cancer cases with known SHS exposure from parents during childhood in a population of never-smokers. This allowed us to focus on the breast-cancer risk among never-active-smoking women, which describes most women globally. Another strength is that we were able to estimate the burden of breast cancer based on individual exposure data and not on country prevalence. Also, the study population is representative of the Norwegian middle-aged female population, according to both smoking exposure^43^^,^^44^ and breast-cancer incidence,^19^ and reflects known smoking patterns^3^ and breast-cancer incidences for Norwegian women.^2^

We consider it a strength that we were able to address four (exposure at an early age/before first pregnancy, the extent to which the use of alcohol confounds the association, the risk according to menopausal status and the risk according to oestrogen hormone-receptor status) of the seven research questions on smoking and breast-cancer risk listed in the Surgeon General's report^10^ and that the association between SHS exposure and breast-cancer risk was materially the same.

Furthermore, we have virtually complete follow-ups through the National Population-based registries. Since our youngest enrolment age was 35 years, it is unlikely that the women have started to smoke during follow-up, as very few women initiate smoking after this age.^33^^,^^45^

The main limitation of the present study is the lack of detailed information, as calendar time, duration and intensity, regarding the SHS exposure from parents. Moreover, we had information on current SHS exposure from spouses at enrolment for fewer than half of the women. We decided to include them in the reference group, which may have attenuated our results. Furthermore, we did not define what we meant by ‘childhood’ in our questionnaires. There may be some residual confounding due to the examined factors or to other factors that we did not measure.

## Biological plausibility

The previously cited expert reports^7–10^ have described the biologic mechanisms by which cigarette smoke may be a cause of breast cancer. There is an agreement that these mechanisms provide plausibility to the causal nature of a smoking exposure–breast cancer association.^7–10^ As emphasized by Colditz *et al.*^46^ in 2014 and underscored in the 2020 World Cancer Report,^11^ the key message in breast-cancer research and prevention efforts is a lifetime approach.

The women in our study were born between 1927 and 1957. The youngest women in our cohort were teenagers in the 1970s—a decade with no general concern about possible negative health effects from SHS exposure. In 2005, the WHO Framework Convention on Tobacco Control entered into force.^47^ As of 2018, overall 91% (*n = *165) of all parties had implemented some measures to protect people from tobacco smoke (Article 8). However, most countries had not banned smoking in private homes or private vehicles when children are present.^48^

In conclusion, we observe that in a population of never-smoking women, those exposed to SHS from parents during childhood had a higher risk of breast cancer than those who were not. Our results suggest that in women exposed, 1 in 10 and, in the population of never-smokers, 1 in 14 breast-cancer cases could have been avoided in the absence of SHS from parents during childhood. We note that the global cancer burden due to SHS may be underestimated.

## Ethics approval

The study was approved by the Regional Committee for Medical and Health Research Ethics (REK NORD).

## Funding

None.

## Data availability

Data available on request. The data underlying this article will be shared upon reasonable request to the corresponding author.

## References

[1] Sung H , Ferlay J, Siegel RL et al Global cancer statistics 2020: GLOBOCAN estimates of incidence and mortality worldwide for 36 cancers in 185 countries. CA Cancer J Clin 2021;71:1–41. 10.3322/caac.21660 33538338

[2] Cancer Registry of Norway. Cancer in Norway 2018—Cancer Incidence, Mortality, Survival and Prevalence in Norway. Oslo: Cancer Registry of Norway, 2019.

[3] Lund I , Lund KE. Lifetime smoking habits among Norwegian men and women born between 1890 and 1994: a cohort analysis using cross-sectional data. BMJ Open 2014;4:e005539. 10.1136/bmjopen-2014-005539 PMC4202009 25326209

[4] Statistisk sentralbyrå [Statistics Norway]. Smoking in Norway, 2018. Oslo: Statistisk sentralbyrå, 2019.

[5] GBD 2015 Tobacco Collaborators. Smoking prevalence and attributable disease burden in 195 countries and territories, 1990–2015: a systematic analysis from the Global Burden of Disease Study 2015. Lancet 2017;389:1885–906. 28390697 10.1016/S0140-6736(17)30819-X PMC5439023

[6] U.S. National Cancer Institute and World Health Organization. The Economics of Tobacco and Tobacco Control. National Cancer Institute Tobacco Control Monograph 21. NIH Publication No. 16-CA-8029A. Bethesda, MD: US Department of Health and Human Services, National Institutes of Health, National Cancer Institute; and Geneva: World Health Organization, 2016.

[7] California Environmental Protection Agency's (CalEPA). Proposed Identification of Environmental Tobacco Smoke as a Toxic Air Contaminant. Part B: Health Effects. Sacramento, CA: California Environmental Agency, Office of Environmental Health Hazard Assessment, 2005.

[8] Johnson KC , Miller AB, Collishaw NE et al Active smoking and secondhand smoke increase breast cancer risk: the report of the Canadian Expert Panel on Tobacco Smoke and Breast Cancer Risk (2009). Tob Control 2011;20:e2. 10.1136/tc.2010.035931 21148114

[9] International Agency for Research on Cancer. International Agency for Research on Cancer (IARC) Monographs on the Evaluation of Carcinogenic Risks to Humans. Vol 100 E.: A Review of Human Carcinogens: Personal Habits and Indoor Combustions. Lyon: International Agency for Research on Cancer, 2012. PMC4781577 23193840

[10] U.S. Department of Health and Human Services. The Health Consequences of Smoking—50 Years of Progress. A Report of the Surgeon General. Atlanta, GA: US Department of Health and Human Services, 2014.

[11] Wild CP , Weiderpass E, Steward BW (eds). World Cancer Report 2020: Cancer Research for Cancer Prevention. Lyon: The International Agency for Research on Cancer, 2020. ISBN (Pdf) 978–92-832–0448-0. 39432694

[12] Glantz SA , Johnson KC. The surgeon general report on smoking and health 50 years later: breast cancer and the cost of increasing caution. Cancer Epidemiol Biomarkers Prev 2014;23:37–46. 24420985 10.1158/1055-9965.EPI-13-1081

[13] Gray JM , Rasanayagam S, Engel C, Rizzo J. State of the evidence 2017: an update on the connection between breast cancer and the environment. Environ Health 2017;16:94. 28865460 10.1186/s12940-017-0287-4 PMC5581466

[14] Macacu A , Autier P, Boniol M, Boyle P. Active and passive smoking and risk of breast cancer: a meta-analysis. Breast Cancer Res Treat 2015;154:213–24. 26546245 10.1007/s10549-015-3628-4

[15] Kim AS , Ko HJ, Kwon JH, Lee JM. Exposure to secondhand smoke and risk of cancer in never smokers: a meta-analysis of epidemiologic studies. IJERPH 2018;15:1981. 10.3390/ijerph15091981 PMC6164459 30208628

[16] Gram IT , Little MA, Lund E, Braaten T. The fraction of breast cancer attributable to smoking: the Norwegian women and cancer study 1991-2012. Br J Cancer 2016;115:616–23. 27280631 10.1038/bjc.2016.154 PMC4997535

[17] Jha P. Avoidable global cancer deaths and total deaths from smoking. Nat Rev Cancer 2009;9:655–64. 19693096 10.1038/nrc2703

[18] Lund E , Kumle M, Braaten T et al External validity in a population-based national prospective study--the Norwegian Women and Cancer Study (NOWAC). Cancer Causes Control 2003;14:1001–08. 14750540 10.1023/b:caco.0000007982.18311.2e

[19] Lund E , Dumeaux V, Braaten T et al Cohort profile: the Norwegian Women an Cancer Study--NOWAC--Kvinner og kreft. Int J Epidemiol 2008;37:36–41. 17644530 10.1093/ije/dym137

[20] Therneau TM , Grambsch P. Modeling Survival Data: Extending the Cox Model. New York: Springer-Verlag, Inc., 2001.

[21] Lau B , Cole SR, Gange SJ. Competing risk regression models for epidemiologic data. Am J Epidemiol 2009;170:244–56. 19494242 10.1093/aje/kwp107 PMC2732996

[22] Austin PC , Lee DS, Fine JP. Introduction to the analysis of survival data in the presence of competing risks. Circulation 2016;133:601–09. 26858290 10.1161/CIRCULATIONAHA.115.017719 PMC4741409

[23] Kleinbaum DG. Survival Analysis: A Self-Learning Text. New York, NY: Springer, 1996.

[24] Vittinghoff E , Glidden DV, Shiboski SC, McCullough CE. Regression Methods in Biostatistics: Linear, Logistic, Survival, and Repeated Measures Models. New York, NY: Springer Science + Business Media, LLC, 2012.

[25] Newson R. *PUNAF: Stata Module to Compute Population Attributable Fractions for Cohort Studies Statistical Software Components S457193*. Boston, MA, USA: Boston College Department of Economics, 2010.

[26] Pirie K , Beral V, Peto R, Roddam A, Reeves G, Green J, Million Women Study Collaborators. Passive smoking and breast cancer in never smokers: prospective study and meta-analysis. Int J Epidemiol 2008;37:1069–79. 18544575 10.1093/ije/dyn110

[27] Reynolds P , Goldberg D, Hurley S et al Passive smoking and risk of breast cancer in the California teachers study. Cancer Epidemiol Biomarkers Prev 2009;18:3389–98. 19959687 10.1158/1055-9965.EPI-09-0936 PMC2908531

[28] Xue F , Willett WC, Rosner BA, Hankinson SE, Michels KB. Cigarette smoking and the incidence of breast cancer. Arch Intern Med 2011;171:125–33. 21263102 10.1001/archinternmed.2010.503 PMC3131146

[29] Luo J , Margolis KL, Wactawski-Wende J et al Association of active and passive smoking with risk of breast cancer among postmenopausal women: a prospective cohort study. BMJ 2011;342:d1016. 21363864 10.1136/bmj.d1016 PMC3047002

[30] Rosenberg L , Boggs DA, Bethea TN, Wise LA, Adams-Campbell LL, Palmer JR. A prospective study of smoking and breast cancer risk among African-American women. Cancer Causes Control 2013;24:2207–15. 24085586 10.1007/s10552-013-0298-6 PMC3855285

[31] Dossus L , Boutron-Ruault MC, Kaaks R et al Active and passive cigarette smoking and breast cancer risk: results from the EPIC cohort. Int J Cancer 2014;134:1871–88. 24590452 10.1002/ijc.28508

[32] White AJ , D’Aloisio AA, Nichols HB, DeRoo LA, Sandler DP. Breast cancer and exposure to tobacco smoke during potential windows of susceptibility. Cancer Causes Control 2017;28:667–75. 28523418 10.1007/s10552-017-0903-1 PMC5530373

[33] Bjerkaas E , Parajuli R, Weiderpass E et al Smoking duration before first childbirth: an emerging risk factor for breast cancer? Results from 302,865 Norwegian women. Cancer Causes Control 2013;24:1347–56. 23633026 10.1007/s10552-013-0213-1 PMC3675272

[34] Gaudet MM , Gapstur SM, Sun J, Diver WR, Hannan LM, Thun MJ. Active smoking and breast cancer risk: original cohort data and meta-analysis. J Natl Cancer Inst 2013;105:515–25. 23449445 10.1093/jnci/djt023

[35] Nyante SJ , Gierach GL, Dallal CM et al Cigarette smoking and postmenopausal breast cancer risk in a prospective cohort. Br J Cancer 2014;110:2339–47. 24642621 10.1038/bjc.2014.132 PMC4007228

[36] Catsburg C , Miller AB, Rohan TE. Active cigarette smoking and risk of breast cancer. Int J Cancer 2015;136:2204–09. 25307527 10.1002/ijc.29266

[37] Gram IT , Park SY, Kolonel LN et al Smoking and risk of breast cancer in a racially/ethnically diverse population of mainly women who do not drink alcohol: the MEC study. Am J Epidemiol 2015;182:917–25. 26493265 10.1093/aje/kwv092 PMC4836396

[38] Gaudet MM , Carter BD, Brinton LA et al Pooled analysis of active cigarette smoking and invasive breast cancer risk in 14 cohort studies. Int J Epidemiol 2016;46:881–93. 10.1093/ije/dyw288 PMC5837778 28031315

[39] Andersen ZJ , Jorgensen JT, Gron R, Brauner EV, Lynge E. Active smoking and risk of breast cancer in a Danish nurse cohort study. BMC Cancer 2017;17:556. 28830370 10.1186/s12885-017-3546-4 PMC5568272

[40] Jones ME , Schoemaker MJ, Wright LB, Ashworth A, Swerdlow AJ. Smoking and risk of breast cancer in the Generations Study cohort. Breast Cancer Res 2017;19:118. 29162146 10.1186/s13058-017-0908-4 PMC5698948

[41] Gram IT , Park SY, Maskarinec G, Wilkens LR, Haiman CA, Le Marchand L. Smoking and breast cancer risk by race/ethnicity and oestrogen and progesterone receptor status: the Multiethnic Cohort (MEC) study. Int J Epidemiol 2019;48:501–11. 30668861 10.1093/ije/dyy290 PMC6469313

[42] Johnson KC , Glantz SA. Evidence secondhand smoke causes breast cancer in 2005 stronger than for lung cancer in 1986. Prev Med 2008;46:492–96. 18182169 10.1016/j.ypmed.2007.11.016 PMC2737483

[43] Gram IT , Braaten T, Lund E, Le Marchand L, Weiderpass E. Cigarette smoking and risk of colorectal cancer among Norwegian women. Cancer Causes Control 2009;20:895–903. 19274482 10.1007/s10552-009-9327-x PMC2694321

[44] Gram IT , Sandin S, Braaten T, Lund E, Weiderpass E. The hazards of death by smoking in middle-aged women. Eur J Epidemiol 2013;28:799–806. 24078008 10.1007/s10654-013-9851-6 PMC3824303

[45] Gram IT , Braaten T, Terry PD et al Breast cancer risk among women who start smoking as teenagers. Cancer Epidemiol Biomarkers Prev 2005;14:61–66. 15668477

[46] Colditz GA , Bohlke K, Berkey CS. Breast cancer risk accumulation starts early: prevention must also. Breast Cancer Res Treat 2014;145:567–79. 24820413 10.1007/s10549-014-2993-8 PMC4079839

[47] World Health Organization. WHO Framework Convention on Tobacco Control Deadline Approaches. Geneva: World Health Organization, 2003.

[48] World Health Organization. 2018 Global Progress Report on the Implementation of the WHO Framework Convention on Tobacco Control. Geneva: World Health Organization, 2018.

